# The Efficacy of Complementary and Alternative Medicine in the Treatment of Female Infertility

**DOI:** 10.1155/2021/6634309

**Published:** 2021-04-23

**Authors:** Jiaxing Feng, Jing Wang, Yuehui Zhang, Yizhuo Zhang, Liyan Jia, Dongqi Zhang, Jiao Zhang, Yanhua Han, Shoujuan Luo

**Affiliations:** ^1^Heilongjiang University of Chinese Medicine, Harbin, China; ^2^People's Hospital of Langfang City, Langfang, China; ^3^Department of Obstetrics and Gynecology, First Affiliated Hospital, Heilongjiang University of Chinese Medicine, Harbin, China; ^4^Department of Acupuncture and Moxibustion, Second Affiliated Hospital, Heilongjiang University of Chinese Medicine, Harbin, China; ^5^Digestive Hospital of Heilongjiang Hospital, Harbin, China

## Abstract

Female infertility is a state of fertility disorder caused by multiple reasons. The incidence of infertility for females has significantly increased due to various factors such as social pressure, late marriage, and late childbirth, and its harm includes heavy economic burden, psychological shadow, and even marriage failure. Conventional solutions, such as hormone therapy, in vitro fertilization (IVF), and embryo transfer, have the limitations of unsatisfied obstetric outcomes and serious adverse events. Currently, complementary and alternative medicine (CAM), as a new treatment for infertility, is gradually challenging the dominant position of traditional therapies in the treatment of infertility. CAM claims that it can adjust and harmonize the state of the female body from a holistic approach to achieve a better therapeutic effect and has been increasingly used by infertile women. Meanwhile, some controversial issues also appeared; that is, some randomized controlled trials (RCTs) confirmed that CAM had no obvious effect on infertility, and the mechanism of its effect could not reach a consensus. To clarify CAM effectiveness, safety, and mechanism, this paper systematically reviewed the literature about its treatment of female infertility collected from PubMed and CNKI databases and mainly introduced acupuncture, moxibustion, and oral Chinese herbal medicine. In addition, we also briefly summarized psychological intervention, biosimilar electrical stimulation, homeopathy, hyperbaric oxygen therapy, etc.

## 1. Introduction

The World Health Organization defines infertility as a failure of a couple to conceive after one year of regular unprotected intercourse. Nearly 15% of couples of childbearing ages worldwide suffer from infertility, most of whom are residents of developing countries [1, 2]. Nearly 30% of infertility factors are associated with males, about 40% with both males and females, and approximately 20–70% with females [3, 4]. Women, therefore, play more important roles in infertility. The primary causes of female infertility usually include ovulation disorders, fallopian tube problems, uterine lesions, and endometriosis [5]. The conventional treatments of infertility include sex hormone therapy (follicle-stimulating hormone, human chorionic gonadotropin, etc.), tubal plastic surgery, and assisted reproductive technology. These therapies, however, have unavoidable side or/and adverse effects. Hormone therapy, for example, can give rise to ovarian hyperstimulation syndrome (OHSS) or mental illness [6, 7]. IVF was initially utilized as an assisted reproductive technology to solve tubal obstruction and now is used for treating infertility. It has been 42 years since the first IVF baby was born in 1978 [8]. Although the live rate of embryo transfer has increased during the past years, the result is still lower than the expectation of patients. Meanwhile, its extremely high cost also makes most infertile couples in the world unable to afford it [9].

CAM called “Unconventional Medicine” or “Unorthodox Medicine” covers various treatments, including not only traditional medicine and folk therapies but also many new therapies that cannot be covered by medical insurance. CAM treatments consist of Chinese medicine (Chinese herbal medicine, acupuncture, and moxibustion, Qigong), Indian medicine, medicinal foods, health foods, aromatherapy, vitamin therapy, diet therapy, psychotherapy, spa, oxygen therapy, etc. CAM is a commonly used adjuvant therapy widely accepted by infertility patients. Some RCTs have found that these interventions are helpful for the conception of infertility patients [10, 11]. Although there are many overviews on CAM treatment for infertility, its therapeutic effect and mechanism are still controversial. This review summarizes CAM treatment of infertility and briefly generalizes its mechanism.

## 2. Overview of Acupuncture and Moxibustion Treatment of Infertility

Acupuncture and moxibustion, an essential part of Traditional Chinese Medicine (TCM), have been protecting the health of the Chinese people for five thousand years. As a symbol of TCM, it is being accepted by most countries in the world. Guided by meridian and acupoint theory, acupuncturists take the human body as a whole and employ acupuncture and moxibustion, a unique clinical technique, to treat and prevent diseases. This technique uses needles and Artemisia as tools and raw materials, through inserting the needles and burning the leaves to stimulate the specific parts of the body, to adjust the balance of the body for disease treatment and prevention [12–14]. This therapy can be traced back at least 3000 years ago. With the modernization of TCM, acupressure, electroacupuncture (EA), moxibustion, and laser acupuncture have branched out from the original model. At present, about 180 countries around the world apply acupuncture and moxibustion to the treatment and prevention of diseases, out of which more than 50 countries consider acupuncture and moxibustion as CAM [15].

In recent years, acupuncture and moxibustion have become ideal treatments for infertility due to many of their superiorities. Because of the complex etiology, the treatment of infertility commonly takes a long time and the success rate is relatively low. The effect of acupuncture and moxibustion, however, is rapid and significant, because they can reinforce body function and improve the disease resistance of the body. Meanwhile, they are easy to operate and economical. Besides, there are fewer side effects, which can be avoided by careful operation. Furthermore, they can also be used as adjuvant therapy in combination with conventional therapies. The efficacy has aroused the interest of many clinicians and medical scientists. Clinical or animal studies have been conducted to evaluate the effect of acupuncture and moxibustion on infertility and papers have been published to elaborate the mechanism. However, the results across studies varied widely, some RCTs show that the treatment for infertility is beneficial, while others indicate otherwise. And the mechanism of acupuncture and moxibustion for infertility is still controversial.

### 2.1. The Application of Acupuncture in Infertility

Acupuncture is one of the most studied CAM interventions that were related to the improvement of reproductive outcomes [16]. Acupuncture has a long history of treating gynecological diseases. An increasing number of researches have indicated that acupuncture can regulate menstruation and assist female pregnancy without the risk of multiple pregnancies [17, 18]. According to The Yellow Emperor's Inner Classic: The Spiritual Pivot Nine Needles and Twelve Source Points: “The most crucial thing in acupuncture is to get the needling sensation. When it presents, the curative effect will be better” [19]. Needling sensation, also known as obtaining qi, refers to the feeling that needles are punctured into acupoints and apply with manipulation. It usually manifests as soreness, numbness, pain, and other reactions. Furthermore, better efficacy can be achieved by giving stimulation after obtaining qi [20]. Clinical practice showed that different interventions can produce different amounts of stimulus, which directly affected clinical efficacy [21]. Manual acupuncture and EA are currently the most popular acupuncture protocols. Manual acupuncture refers to rotating the needles with fingers, while electroacupuncture is the combination of acupuncture and electrical stimulation, both of them aiming to increase the therapeutic effect. There is no research to show which stimulus is more effective for reproductive function [22].

### 2.2. Clinical Effect of Acupuncture on Infertility

Most western countries' cognition of acupuncture came after President Nixon visited China in the 1970s. Since then, a completely new understanding of TCM was gained in western countries, and the remarkable effect of acupuncture in treating diseases fascinated western practitioners [23]. Although acupuncture has been accepted for treating ache, it has no substitute for anesthesia [24]. In recent years, acupuncture has been increasingly utilized as an auxiliary method for infertility and has been widely used in various circumstances during pregnancy. However, from the perspective of physiology, it is difficult to reach a consensus on the mechanism of acupuncture in the treatment of diseases [25]. Before the 21^st^ century, there were few reports on the research of acupuncture in reproductive medicine, especially large-sample RCT. In 2002, Paulus et al. carried out an RCT, which reported the effect of acupuncture on the pregnancy rate of IVF for the first time. The 160 recruited patients randomized to the control group and the acupuncture group received acupuncture treatment 25 minutes before and after embryo transfer. Compared with the control group, the pregnancy rate in the acupuncture group significantly increased (42.5% versus 26.3%; *P* < 0.03) [26]. Subsequently, in 2006, Stefan Dieterle et al. conducted an RCT to investigate the effect of acupuncture during the luteal phase on IVF/ICSI outcomes. 225 infertile patients were randomized to the treatment group and the control group. In the treatment group, acupuncture was performed in line with the principles of TCM, while the control group chose the placebo acupoints for comparison. The results showed that the pregnancy rate and implantation rate in the treatment group were 29.4% and 12.6%, respectively, while those in the control group were 8.2% and 3.2% (*P* < 0.01), which concluded that acupuncture in the luteal phase has a positive effect on IVF/ICSI [27]. Recently, LY et al. conducted a clinical trial to observe the effect of acupuncture combined with Chinese medicine on infertility patients with thin endometrium. 60 patients were randomized to the treatment group receiving acupuncture combined with Chinese medicine and the control group received estradiol valerate tablets. The results showed that endometrium-thickness and pregnancy rate in the treatment group were significantly higher than those of the control group, and the difference was statistically significant (*P* < 0.05). Therefore, it can be concluded that acupuncture combined with Chinese medicine can promote the growth of endometrium and improve the clinical pregnancy rate [28]. With more positive reports, acupuncture for infertility is gradually accepted by countries all over the world. In the past few years, several meta-analyses on acupuncture for infertility have been published. Although these studies have different degrees of bias risk, the conclusions still add weight to acupuncture as a substitution for western medical therapy. Among them, a meta-analysis was conducted to evaluate the efficacy of acupuncture or clomiphene (CC) or acupuncture combined with CC in treating anovulatory infertility for the first time. Compared with CC, acupuncture had better treatment results in pregnancy rate and maximum follicle diameter. At the same time, this meta-analysis also pointed out that CV3, CV4, CV6, ST36, SP6, and EX-CA1 are the most commonly selected for treating anovulatory infertility women, and it suggests that the above acupoints should give priority in future treatment [29].

While numerous articles have reported the positive effect of treating infertility with acupuncture, some scholars still questioned and denied the efficacy. In the acupuncture RCT, sham acupuncture or placebo acupuncture is usually the control group, which is needling on nonacupoint. Some scholars believe that even stimulating nonacupoints can produce therapeutic effects [21, 23]. In 2017, the team of Professor Wu published an article on JAMA entitled “Effect of Acupuncture and Clomiphene in Chinese Women with Polycystic Ovary Syndrome: A Randomized Clinical Trial.” This is a multicenter RCT to explore whether acupuncture or acupuncture combined with CC can increase the fertility rate of women with PCOS. The results showed that there was no significant difference in the live birth rate between active acupuncture and control acupuncture (29.4% versus 28.0%, 13.9% versus 16.8%). Finally, the conclusion is as follows: “compared with acupuncture plus placebo, acupuncture with or without CC could not improve the live birth rate of Chinese women with PCOS.” Therefore, using acupuncture for infertility in such patients was not supported [30, 31]. The research results of Madaschi et al. showed that giving acupuncture immediately before and after embryo transfer did not affect the outcome in general [32]. The results of Rashidi et al. showed that although acupuncture did not affect the IVF/ICSI results of women with PCOS, it has a beneficial efficacy on embryo quality at the early stage of oocyte recruitment. Further research is needed to prove how to transform the improvement of embryo quality into a high pregnancy rate. Multiple systematic reviews and meta-analyses have shown that there is insufficient evidence to support acupuncture in promoting live birth, pregnancy, and ovulation [33]. However, it found that acupuncture can promote the recovery of the menstrual cycle and reduce the levels of luteinizing hormone and testosterone in patients with PCOS [34].

The reasons for these different results may be related to the heterogeneity of clinical trials such as the experience of acupuncturists, the selection and positioning of acupoints, whether to use electrical stimulation or other manipulations, and the course of treatment. Other possible reasons, such as fewer subjects and non-RCT, may also lead to different results. Therefore, shortly, some larger sample, prospective, double-blind, placebo-controlled RCTs are urgently needed to clarify it. We have listed some RCTs in Table 1.

### 2.3. Mechanism of Acupuncture in Treating Infertility

Studies have shown that acupuncture can induce reactions that activate nerve, endocrine, and immune signaling pathways by inserting the skin [35]. The possible mechanisms are listed as follows. First of all, acupuncture makes the gonadotropin (GN) and steroid hormone cycles work together via the hypothalamic-pituitary-ovarian axis (HPOA) to promote the selection of dominant follicles and prepare for embryo implantation. Secondly, acupuncture can improve abnormal ovarian perfusion and the state of diminished ovarian reserve and enhance the quality of oocytes. Finally, acupuncture provides suitable conditions for embryo implantation by improving endometrial morphology, promoting endometrial microcirculation, and regulating estrogen and progesterone receptors in both directions [36–38]. Now, we will discuss these aspects.

#### 2.3.1. Acupuncture Regulates Hypothalamic Function

The hypothalamus regulates the release of luteinizing hormone (LH) and follicle-stimulating hormone (FSH) by secreting gonadotropin-releasing hormone (GnRH), thus controlling the secretion of estrogen and progesterone [36]. Under the action of GN, the ovaries ovulate periodically, accompanied by cyclical secretion of E and P. By regulating the release of hypothalamic neurotransmitters and the secretion of GnRH and GN, acupuncture can improve the abnormal function of HPOA in infertile patients and restore the menstrual cycle, ovulation, and fertility [39]. Studies have shown that acupuncture can regulate the production and secretion of inhibitory neurotransmitters, including dopamine, gamma-aminobutyric acid, and *β*-endorphin (*β*-EP). They have an inhibitory effect on hypothalamic activity, among which *β*-EP is one of the main inhibitors. *β*-EP directly inhibits GnRH neuron activity by binding to its receptor, thereby inhibiting GnRH secretion. Stener-Victorin et al.'s research on animals and patients with PCOS pointed out that acupuncture could regulate the production and secretion of central and peripheral *β*-EP, thereby affecting the release of GnRH and GN [40]. Acupuncture has a bidirectional regulation effect. It can also regulate the release of excitatory neurotransmitters, including leptin and glutamate, to stimulate the function of the hypothalamus. Kamyabi et al. proposed that high leptin might harm the internal environment required for ovarian function and embryonic development, which could be one cause of infertility [41, 42]. Meanwhile, a study has found that obese mice are infertile due to low leptin, and their reproductive function has been improved after leptin injection [43]. After six weeks of auricular point intervention by Hsu CH, they found that the level of leptin was significantly reduced [44]. Therefore, acupuncture can effectively adjust the HPOA function.

#### 2.3.2. Acupuncture Regulates Ovarian Function

Apart from regulating the HPOA, acupuncture can also directly affect the ovary and other peripheral tissues [45–47]. Research conducted by Julia Johansson et al. showed that repeated acupuncture results in a higher ovulation frequency in lean/overweight women with PCOS [48]. The main function of the ovarian artery is to provide nutrients and transmit related hormones to the ovary. Ovarian artery blood mainly supplies nutrients needed for the growth and development of follicles, so it will directly affect the growth of follicles. The research conducted by Manni et al. suggested that the effectiveness of EA in the regulation of ovarian responsiveness indicates that EA could be an alternative approach to preventing and/or overcoming sympathetic-related anovulation in women with PCOS [49]. The study done by Dolz et al. pointed out that the main cause of anovulation in PCOS patients was the decreased vascular resistance index of the ovarian interstitium and increased interstitial blood vessels [50]. Low-frequency EA at proximal and distal acupoints has proven to be effective in promoting ovulation and increasing the possibility of pregnancy [51]. It can improve the angiogenesis of ovarian vessels and antral follicles. An experiment proved that EA promoted the angiogenesis of antral follicles in PCOS-like rats induced by DHT, thus promoting follicular maturation, ovulation, and luteal formation [52]. Stener-Victorin et al. designed a rat experiment to determine whether EA can increase ovarian blood flow. They found that the change of ovarian blood flow depended on the acupoint and frequency [51, 53]. Besides, acupuncture can affect the secretion of AMH. AMH is secreted by granulosa cells and participates in the development of follicles. Sverre C Christiansen et al. confirmed that anti-Müller's hormone was positively correlated with follicle count and seemed to be a reliable indicator for predicting follicle count [54]. The study of Shi et al. has demonstrated that EA improved follicular arrest by decreasing the excessive expression of AMH to regulate FSH and AMH imbalance in granulosa cells in PCOS [55].

#### 2.3.3. Acupuncture Regulates Uterine Function

Finally, acupuncture can improve the endometrial morphology, promote the microcirculation of the endometrium, and bidirectionally regulate the estrogen and its receptor, which provide good conditions for embryo implantation and improve the pregnancy rate. Endometrial receptivity (ER) refers to the ability of the endometrium to accept the embryo implantation changing with menstruation. ER correlates with infertility, and a good ER is a prerequisite for blastocyst implantation [56]. Embryo implantation is closely related to endometrial thickness, morphology, and blood supply [57]. The thin endometrium is one of the most critical factors for low ER and low pregnancy rates [58, 59]. The endometrium usually divides into three types: A, B, and C. The thinner endometrium of type B and C is not conducive to embryo implantation and development, while type A with a thickness greater than 8 mm is more suitable for embryo implantation and development [60, 61]. Studies have shown that acupuncture can change the type of endometrium, and after treatment, the percentages of type A and B are higher than before. Li selected LI4, LR3, KI3, SP6, and other acupoints to treat IVF-ET patients. The results showed that compared with the control group, acupuncture could increase the endometrial thickness and the pregnancy rate [62]. It can be seen that acupuncture can improve the morphology of the endometrium and can also increase the thickness of the endometrium and the clinical pregnancy rate. The blood supply of the endometrium includes the uterine artery, endometrial, and endometrial blood flow [63]. Studies have shown that reducing the blood flow impedance of bilateral uterine arteries and endometrium can significantly improve the blood flow parameters of the uterine artery [64, 65] and increase uterine blood flow and endometrial thickness, thereby improving ER. It has a positive impact on embryo implantation rate and clinical pregnancy rate [66, 67]. Steer et al. found that when the uterine artery pulsatility index >3.0, the pregnancy rate would be decreased [68]. Meanwhile, Stener-Victorin et al. confirmed that the uterine artery pulsatility index decreased after a series of acupuncture treatments [69]. The results of Ho et al. also confirmed that the pulsatility index of the uterine artery in the acupuncture group was significantly reduced [70]. Besides, acupuncture can also regulate estrogen and progesterone and their receptors. The endometrium is the main target organ of estrogen and progesterone. An appropriate amount of them is conducive to pregnancy. However, the imbalanced ratio of estrogen and progesterone can decrease ER and cause blastocyst-implantation failure. The result of Mu et al. predicted that acupuncture could increase ER and proposed that the potential molecules promoting ER were HSA-Mir-449a, HSA-Mir-3135b, and HSA-Mir-345-3p [38].

### 2.4. Safety of Acupuncture in the Treatment of Infertility

It is well known that acupuncture is relatively safe with fewer adverse events compared with western medicine. Most of them are transient, such as skin erythema, bruising, bleeding, and pain, which can be avoided by careful manipulation; in addition, the reports of serious complications are rarely [71, 72]. In an RCT of IVF, 152 women had adverse events; all of them were mild discomfort or bruises [73]. In another clinical trial involving more than 200,000 patients receiving acupuncture for ache, the incidence of adverse events was only 8.6%. In short, adverse events do occur in acupuncture, but to a large extent, they are mostly minor compared with nonacupuncture-related interventions [74]. In a word, although the existing studies show that acupuncture has a positive effect on infertility and its mechanism is relatively explicit; there are still some limitations, such as insufficient sample size and lack of high-quality evidence in the existing studies. Therefore, more large-scale RCTs are needed to clarify the efficacy and mechanism of acupuncture in infertility.

## 3. Clinical Efficacy and Mechanism of Moxibustion on Infertile Women

Moxibustion refers to burning or fumigating acupoints or lesions by moxibustion to prevent diseases. From the perspective of TCM, moxibustion has the functions of warming meridians and dispersing cold, strengthening the body, and eliminating diseases. It is commonly used in treating infertility, dysmenorrhea, premature ovarian failure, and other gynecological diseases [75–78]. Heat-sensitive moxibustion and drug-separated moxibustion on the umbilicus are commonly used in treating infertility. Heat-sensitive moxibustion is to use the ignited moxa stick to produce a heat-sensitive effect to the heat-sensitive acupoints, which can go directly to the disease site, promote local pelvic blood circulation, accelerate local drug absorption, improve hydrosalpinx to restore its function, improve endometrial thickness, and regulate endometrial receptivity [79, 80]. Several researchers used heat-sensitive moxibustion combined with TCM decoction or western medicine to treat PCOS infertility patients. The results showed that the intervention group was significantly better than the control group in reducing ovarian volume, improving endometrial thickness, reducing LH and T, and increasing E2 and pregnancy rate, which may be related to the decrease of NF-*κ*B and TNF-a [81, 82]. Heat-sensitive moxibustion also has a significant effect on infertility resulting from ovulation disorder and hydrosalpinx [83, 84]. Some researchers use drug-separated moxibustion on the umbilicus combined with CC in treating ovulation disorder infertility; the result showed that the maximum follicle diameter and the endometrial thickness could be significantly increased. The total effective rate, clinical cure rate, estradiol and progesterone levels, and TCM syndrome scores have been greatly improved in luteal insufficiency infertility treated with drug-separated moxibustion on umbilicus combined with oral Chinese medicine [85, 86]. In the theory of TCM, the umbilicus is RN8, which is exterior-interior related to the Du Channel. Moxibustion can invigorate the deficiency and warm the yang and dispel cold while applying moxibustion across the medicine powder for warming the kidney and promoting yang can increase the power of promoting yang. It can be seen that the medicine-separated moxibustion on the umbilicus is an organic combination of moxibustion, acupoints, and drugs. Besides, some studies have shown that moxibustion can inhibit ovarian cell apoptosis and enhance antioxidant defense capacity to improve ovarian function [87]. Modern pharmacological studies have shown that the effective components in Artemisia can activate blood vessels, accelerate blood circulation, improve ovarian artery blood supply, and increase ovarian blood flow perfusion, which can significantly improve ovulation rate and pregnancy rate [88–90].

## 4. Overview of Oral Chinese Herbal Medicine (CHM) in the Treatment of Infertility

TCM is by far the most complete, widely used, and influential medical system in the world [91]. The legend of “Shennong tasting hundreds of herbs” dates back to the commune days. Taking CHM orally is also one of the important CAMs for treating infertility. It is guided by the theory of Yin Yang, Five-Phase, Viscera and Bowels, Qi-Blood, Fluid-Humor, etc., and based on the principle of syndrome differentiation and treatment, which provides individualized treatment for infertility. In recent years, with the improvement of the clinical efficacy evaluation system and the development and implementation of scientific research, more studies have proved that CHM has the advantages, such as significant efficacy and high security in treating infertility. The number of infertile couples seeking TCM for infertility (including oral CHM and Chinese herbal diet) is also increasing.

### 4.1. The Application of Oral CHM Administration for Infertility

TCM, a kind of CAM, seems to be a more popular protocol for treating infertile women. A study conducted by Hung YC showed that among 8766 infertile women, 96.17% of them used TCM for the treatment of infertility in addition to conventional therapies. They also noted the female infertile patients who suffered from the diseases such as endometriosis, uterine fibroids, or irregular menstrual cycles were more willing to seek TCM treatment [92]. CHM has a significant effect in treating infertility, which is mainly achieved by improving follicular development, reducing the inflammatory environment of the uterine cavity, and improving hormone levels, etc.

### 4.2. Clinical Effect of Oral CHM Administration for Infertility

Many RCTs showed that administrating conventional therapies combined with oral CHM could greatly improve the ovulation rate, clinical pregnancy rate, etc. of infertile patients. Wan YT et al. randomly assigned 150 infertile patients caused by ovulation disorder into three groups. All three groups were given CC. On this basis, one group was given CHM and the other group was given aspirin. After three menstrual cycles of treatment, the results showed that the ovulation rate and pregnancy rate in the CHM combined with the CC group were significantly higher than the other two groups (*P* < 0.05) [93]. Tian et al. found that compared with the hormone alone, hormone combined with CHM can significantly improve the pregnancy rate (*P* < 0.05) [94]. Modern pharmacological studies have shown that Dodder seed, a kind of CHM, can regulate the function of HPOA, promote the secretion of estrogen and progesterone in rats with embryo implantation dysfunction, and thus improve ER [95]. CHM also has advantages in improving follicular development. Cai et al. found that compared with the group without CHM treatment, CHM treatment can significantly promote follicular development and ovulation (*P* < 0.05) without increasing the incidence of adverse reactions [96]. The blood-activating and stasis-resolving medicine, Sichuan lovage root, in this prescription has the effect of improving the hemorheology and microcirculation of ovary and uterus, facilitating follicular development and ovulation, and improving ER [97]. Liu's experiment also reached the same conclusion by using another prescription [98]. CHM can also treat salpingitis, thereby greatly increasing the fertility rate of patients with infertility caused by fallopian tube factors [99]. Gao administrated CHM to infertile patients caused by chronic pelvic inflammation. The results showed that the combination of western medicine and CHM was better than western medicine alone in improving tubal adhesion, hydrops, pregnancy rate, etc. (*P* < 0.05) [100]. Feng randomized 80 patients with tubal obstructive infertility into the observation group (receiving hydrotubation combined with CHM) and control group (receiving hydrotubation). The results showed that the effective rates of the two groups were 92.50% versus 75.00% respectively, and the symptoms in the observation group were significantly alleviated compared with the control group [101]. Zhai and Lang also confirmed that CHM treatment can improve the inflammatory state, thereby improving the pregnancy rate [102, 103]. Premature ovarian failure is also a major cause of infertility. Premature ovarian failure is also a major cause of infertility. Wang KL studied 56 cases of infertility caused by premature ovarian insufficiency (POI) and found that Bushen Culuan Decoction could effectively improve ovarian reserve in patients with POI [104]. Oral CHM treats infertility by improving hormone levels. Gong et al. divided 80 infertile patients with PCOS into the control group (*n* = 40), and the observation group (*n* = 40) added CHM based on the control group. After treatment, the levels of T and LH in the observation group decreased more significantly than those in the control group (*P* < 0.05), and the pregnancy rate in the treatment group was much higher than that in the control group (85% versus 65%) (*P* < 0.05) [105]. Men found that the efficacy of bromocriptine combined with CHM was significantly better than bromocriptine alone for infertile patients with hyperprolactinemia. After treatment, all indicators in the two groups were improved; however, the efficacy of the combined group was better than the bromocriptine alone (95.0% versus 77.5%) (*P* < 0.05) [106]. Related research showed that the raw germinated barley in this prescription had the effect of reducing prolactin [107]. TCM takes pattern differentiation and treatment as the principle and gives corresponding prescriptions based on patients' pathological states. Zhao et al. conducted a multicenter RCT, which confirmed that taking CHM could significantly improve the pregnancy rate and live birth rate of infertile patients with endometriosis after laparoscopic surgery (*P* < 0.05) [108]. Liu's trial reached the same conclusion and found that the abortion rate could be reduced as well (*P* < 0.01) [109].  Table 2 lists the prescription composition of the above protocols and some protocols not mentioned [110–113].

Most RCTs have shown that CHM is beneficial in treating infertility, but there are also a small number of trials showing that it is ineffective. Lan et al. conducted an RCT on 80 infertile patients with follicular dysplasia. The control group of 40 patients was given CC combined with estradiol valerate, and the treatment group of 40 patients was given CC combined with estradiol valerate additionally CHM orally. There was no significant difference in pregnancy rate between the two groups after treatment [114]. Moreover, Zhou et al. made a systematic review on the treatment of PCOS with oral CHM. But they failed to collect enough high-quality literature to indicate that CHM had a positive effect on the live birth rate of infertile women with PCOS. Although some literature suggested that the addition of CHM to CC might improve the pregnancy rate, due to the small sample size, wide confidence interval, and other reasons, the quality of the literature was low and there was insufficient evidence to demonstrate the absolute safety of CHM [115]. Due to the heterogeneity of the patient's age, etiology of infertility, previous treatments, and different interventions, the results across studies varied widely. Therefore, larger-sample, multicenter, double-blind, placebo-controlled trials are needed to verify the efficacy and safety of oral CHM in infertility treatment in the future.

### 4.3. Mechanisms of CHM in Treating Infertility

#### 4.3.1. CHM Regulates Uterine Function

Improving ER is one of the mechanisms of oral CHM in treating infertility. Taking CHM can increase endometrial thickness and ER through molecular pathways and gene expression changes, thus improving the pregnancy rate. Liu et al. conducted a study on 120 patients. They found the expression of Hox10 mRNA and ER in the CHM group was increased, which suggested that the treatment of CHM improved ER by increasing the expression of Hox10 mRNA in the endometrium [116]. Yang et al. also reached a similar conclusion in the study of SD rats with implantation disorders [117]. Xin et al. studied the effects of CHM on ER and endometrial angiogenesis in rats and concluded that CHM can promote ER recovery and endometrial angiogenesis by regulating the expression of PI3K, HIF-1A signaling pathway, and VEGF [118].

#### 4.3.2. CHM Regulates Ovarian Function

CHM can improve autophagy or apoptosis of ovarian granulocyte and protect ovarian function by regulating the molecular signaling pathway and molecular expression. Gao studied the effect of CHM on follicular development in rats with follicular dysplasia. He thought that it may be through activating PI3K/Akt/mTOR signaling pathway, reducing the apoptosis-related molecule cleaved Caspase-3 and the apoptosis rate of rat ovarian granulosa cells (GCS), which provides a new idea for the treatment of follicular development disorders [119]. Li used a similar rat model to study and found that CHM may increase the expression of IGF-1R and HIF-1A in the rats' ovarian and downregulate the expression of proapoptotic factor FOX03a, to inhibit excessive follicular atresia and to promote the growth and development of follicles [120]. Chen used circrNAS chip technology to screen the differentially expressed circrNAS in plasma of POI patients and normal people. 35 differentially expressed circrNAS were screened and partially differentially expressed circrNAS were verified by quantitative RNA (QRT-PCR). The results showed that HSACirC0000367 was downregulated in POI patients, suggesting that HSACirC0000367 may play an important role in the POI process [121]. Shi and Sun et al. also reached the same conclusion [122, 123].

#### 4.3.3. CHM Decreases Tubal Inflammation

Hydrotubation is often used in tubal obstructive infertility, but the efficacy cannot meet expectations. TCM takes the damp heat and obstructs the uterus as the etiology and pathogenesis of this disease, which leads to the failure of fertilized ovum formation. The treatment is usually based on invigorating blood and dissolving stasis. The treatment of CHM can significantly reduce the inflammatory factors, thus improving the pregnancy rate. Qiu's research found that the mechanism of CHM in treating tubal obstructive infertility may be through regulating the gene of TLR2, MyD88, and NF-*κ*B and inhibiting the NF-*κ*B Effects of serum-containing Tongguan Pill on TLR2, MyD88, and NF-*κ*B gene expression in macrophage inflammatory models [124]. A study has shown that high concentrations of TNF-*α* were detected in the tubal fluid of patients with tubal inflammation infertility, which was believed to be a certain role in the occurrence and development of tubal inflammatory infertility [125]. Ma et al. conducted an RCT on 82 patients with tubal obstructive infertility and found that hysteroscopic tubal fluid drainage combined with CHM could effectively improve the efficacy and significantly reduce the TNF-*α* [126].

#### 4.3.4. CHM Improves Hormone Levels

The endocrine disorder is the main factor leading to infertility. Oral CHM has an obvious effect on improving hormone disorder in infertile patients. Numerous studies have found that oral CHM could improve pregnancy rates by regulating HPOA, improving insulin resistance, etc. Cao DD treated POI rats with CHM and found that it could improve impaired ovarian function and regulate sex hormones mainly through the MAPK pathway [127]. Jiang gave Bushen Cuyun Recipe (BCR) for the DOR rats. After treatment, the ovarian morphology, follicle, corpus luteum, and serum AMH of the DOR rats were significantly improved. Through network pharmacologic analysis, they found that the possible mechanism of BCR for infertility was the regulation of HPOA and prevention of ovarian granulosa cell apoptosis [128]. Zhang administrated CHM to PCOS-like rats and found that this medicine could effectively reduce the weight of rats and improve endocrine disorders [129]. After treatment with Liuwei Dihuang Pills, Qiu ZX found that the polycystic morphology of the ovaries of the PCOS-like rats was significantly restored. The possible mechanism could be the upregulation of CYP19A1 to restore follicular development and PI3K/Akt signaling pathway to reduce insulin resistance [130]. Yao treated the rats with hyperprolactinemia with CHM and found that the symptoms improved significantly. The mechanism may be through increasing the expression of IP3, PKC, and CaMK in the hypothalamus of rats to open the Ca2+ channel, thus further strengthening the signal transduction of dopamine D2 receptor [131].

All in all, the mechanism of oral CHM in treating infertility is pretty complex. To clarify the more accurate mechanism of its treatment of infertility, more research studies are needed in the future to provide uniform and accurate guidance for clinical treatment.

### 4.4. Clinical Efficacy and Mechanism of Chinese Herbal Diet Therapy in Treating Infertility

Food is the foundation of human existence. Since ancient times, there has been a saying in TCM that medicine and food are homologous. In addition, The Yellow Emperor's Inner Classic has clear requirements on the quality, quantity, time, cold or hot food, and compliance with the four seasons [132]. Chinese herbal diet therapy has also played an indispensable part in disease prevention and treatment. In the process of treating disease, corresponding herbal diet therapy is given based on the physical condition and disease pathology of patients [133]. Herbal diet therapy can also treat infertility. For patients who need to take medicine for a long time, adjuvant dietary therapy can reduce the burden of the digestive system [134]. When treating infertility, professor Ban XW usually adds mutton, soybean, duck, sea cucumber, etc. to CHM to increase the efficiency of medicine [135]. When treating infertility, professor Ban XW usually adds mutton, soybean, duck, sea cucumber, etc. to CHM to increase the efficiency of medicine. The theory of “taking the viscera to nourish the viscera” is also a treatment proved by thousands of years' practice. For example, pig liver, chicken liver, and other animal livers cooperated with Chinese herbs to treat infertility caused by liver depression; common yam rhizome, fleeceflower root, etc. to cure infertility of yin deficiency [134, 135].

Kang J treated DOR infertility with CHM and supplemented it with medicinal food, which effectively improved the clinical efficacy [136]. Wang et al. conducted an RCT to investigate the efficacy of the medicinal diet recipe “Warm Uterus Bao” combined with letrozole in the treatment of PCOS ovulatory infertility. After three menstrual cycles, the results showed that the effective rate of the treatment group was 90.00%, much higher than the control group (76.67%), with a statistically significant difference (*P* < 0.05). The ovulation rate and the pregnancy rate of the treatment group and control group were 81.18% versus 47.73% (*P* < 0.01) and 33.33% versus 10.0%, respectively (*P* < 0.05). This indicated that the combination of the medicated diet “Warm Uterus Bao” with letrozole had a better effect in treating PCOS dysfunction infertility, which can effectively improve the symptoms of patients and increase the ovulation rate and pregnancy rate [137]. Huang ZT studied 75 patients with anovulatory infertility and found that sea cucumber could promote endometrial growth, thereby increasing the pregnancy rate [138]. There are few studies on the mechanism of dietary therapy in infertility. Relevant articles discussed that fish, carrots, sesame, walnut, and other foods described in Essentials from the Golden Cabinet are antiaging and longevity [139]. It is hoped that there will be more research studies on the effects of diet therapy on infertility to better guide patients with a healthy diet and play an auxiliary role in treating infertility.

## 5. Overview of Other CAM in the Treatment of Infertility

In addition to acupuncture and moxibustion and oral CHM, there are still other CAMs in treating infertility, such as Chinese medicine enema therapy, psychological intervention, and bionic electrical stimulation.

### 5.1. The Application of TCM Retention Enema in the Treatment of Infertility

The efficacy of TCM retention enema on tubal obstructive infertility is particularly significant. A warm enema containing Chinese medicine is administered before going to bed to treat fallopian tube adhesion. The drug can be absorbed directly by rectal mucosa, which is beneficial to improve the congestion, edema, adhesion, and hyperplasia of local tissues, and thus restoring the function of the fallopian tube [140, 141]. Xu conducted a clinical trial by giving TCM decoction retention enema to patients with tubal infertility, which had obvious efficacy. He believed that the structure of the rectum was close to the uterus, with a large number of venous plexuses and thin walls. Chinese medicine could penetrate the pelvic cavity through venous plexuses, improve the local microenvironment and blood circulation, and reduce inflammatory exuding [142]. Some researchers used TCM retention enema after a hysteroscopy to reduce the levels of TNF-*α*, IL-6, and IL-8, effectively slow down the chronic inflammatory response, improve the patency of fallopian tubes and the abnormal leucorrhea, and lower abdominal pain, thereby promoting the recovery of fertility [143–146]. Some researchers also used the external application of TCM and TCM retention enema after tubal interventional recanalization, which had a significant influence on hemorheology and also promoted the fertility of patients [147]. TCM retention enema combined with acupuncture can also improve the patency of fallopian tubes and pregnancy rates [148, 149]. Some researchers also treated the patients for thin-endometrial infertility with TCM retention enema combined with acupuncture, which improved the blood supply and morphology of the endometrium, enhanced ER, and further increased the pregnancy rate [150]. In addition, for infertility caused by endometriosis, western medicine is prone to relapsing with laparoscopic surgery alone, so it is necessary to use follow-up drugs. Some researchers used TCM retention enema after laparoscopic surgery to improve symptoms such as dysmenorrhea, menorrhagia, and dyspareunia, improve pregnancy rate, and regulate the balance of MMP-9 and TIMP-1 [151]. All studies mentioned above have been listed in Table 3.

### 5.2. The Application of Psychological Interventions on Patients of Infertility

Infertile patients, under pressure from society and family, have an urgent expectation of pregnancy, so they are prone to produce various negative emotions. Patients who particularly failed in IVF-ET have a higher degree of anxiety, which will further lead to reproductive endocrine dysfunction, thus affecting the success rate of treatment [152, 153]. Psychological interventions for infertility include health education, psychological counseling, relaxation training, and mindfulness-based stress reduction. First, it is beneficial to relieve patients' pressure, improve patients' mental health and somatization symptoms, restore fertility function, and increase the success rate of conception [154, 155]. Secondly, psychological intervention can also regulate the mood of infertile patients and improve sleep and life quality, thereby improving the pregnancy outcome of assisted reproductive technology [156, 157]. However, some studies have failed to confirm the effectiveness of the psychological intervention on the pregnancy outcome of assisted reproductive technology, so further studies are needed [158]. Thirdly, studies have confirmed that yoga, a form of relaxation training, can not only regulate physical and mental state and improve ART outcome but also reduce vaginal assisted delivery, relieve pain, and improve fetal outcome [159].

### 5.3. The Application of Biosimilar Electrical Stimulation on Patients of Infertility

Biosimilar electric stimulation can regulate nerve reflex and muscle tension, improve local blood perfusion, and promote tissue regeneration by stimulating pelvic nerve and muscle with various frequency currents. It is usually used to treat postpartum repair, uterine prolapse, urinary incontinence, and infertility. Otherwise, studies have shown that biosimilar electrical stimulation can be used to treat thin-endometrial infertility which can promote endometrial growth, increase blood perfusion, and improve EA and pregnancy rate [160, 161]. Biosimilar electrical stimulation can also accelerate the recovery of ovarian reserve function [162, 163].

### 5.4. The Application of Homeopathy on Patients of Infertility

Homeopathy is to activate the body's ability to heal itself and the immune system to facilitate the recovery process by using a low dose of homeopathic medicine. It is characterized by treating the body as a whole and addressing the causes of the disease rather than focusing on individual symptoms. Homeopathy is effective for infertility caused by psychological problems, ovulation disorders, sperm abnormalities, and unknown causes [164, 165]. Meanwhile, homeopathy is effective in improving patients' health, sperm quality, and hormone levels [166, 167]. Parveen used individualized homeopathy to successfully deliver a healthy newborn in an infertile patient with endometriosis complicated with fallopian tube abnormality and insufficient ovarian reserve, which suggested that homeopathy has a positive effect on infertility [168].

### 5.5. The Application of Hyperbaric Oxygen Therapy on Patients of Infertility

Hyperbaric oxygen therapy can increase blood oxygen content and oxygen partial pressure and improve the state of the whole-body organs. It can be used to treat infertility, acute kidney injury, and wound nonunion of malignant tumor [169]. Studies have shown that hyperbaric oxygen therapy can improve uterine hemodynamics and ER, improve ovum quality, and improve the reproductive capacity of infertile patients [170, 171]. However, studies have shown that hyperbaric oxygen therapy did not promote endometrial thickening but increased serum AMH level, which still needs further research [172].

## 6. Summary

In recent years, infertility has become the third disease after cardio-cerebrovascular disease and tumors. Women with infertility are also at increased risk of developing mental illness. Many patients and doctors are not satisfied with the efficacy of the conventional treatment. CAM, widely accepted as adjuvant therapy for infertility in many Western countries, has met a medical need in the infertile population. However, its effectiveness and safety are still controversial. Acupuncture and moxibustion and CHM are the most commonly used CAM for infertility. Besides, enema therapy and psychological intervention, etc. are also mentioned in this review. At present, there are some limitations with CAM treatment of infertility, such as small sample size, low quality, and lack of uniform standards. Therefore, the validation of CAM's effectiveness has been hindered. Therefore, we look forward to more high-quality studies on CAM in the treatment of infertility.

## Figures and Tables

**Table 1:** Summary of randomized studies of the effect of acupuncture on infertility outcomes.

Study ID	Design	Sample size	Interventions	Outcomes	Limitation
26	RCT	160	Treatment arm: acupuncture intervention control arm: no intervention	Treatment arm: PR, 42.5% [34 of 80]^*∗*^ control arm: PR, 26.3% [21 of 80]	Not mentioned blindness Small sample size

27	Double-blind, RCT	225	Treatment arm: acupuncture intervention control arm: placebo acupuncture intervention	Treatment arm: PR, 33.6%; OPR, 15.6%^*∗*^ control arm: PR, 28.4%; OPR, 13.8%	Small sample size

28	RCT	60	Treatment arm: acupuncture combined with TCM intervention control arm: estradiol valerate tablets intervention	Treatment arm: PR, 26.7% [8 of 30]^*∗*^ control arm: PR, 6.7% [2 of 30]	Not mentioned blindness Small sample size

31	Double-blind, RCT	1000	Treatment arms: active acupuncture plus clomiphene group; active acupuncture plus placebo group control arms: control acupuncture plus clomiphene group; control acupuncture plus placebo group	There were no significant differences in outcomes of LBR between treatment arms and control arms	

34	RCT	62	Treatment arm: acupuncture intervention control arm: no intervention	There were no significant differences in outcomes of OPR between the two groups	Not mentioned blindness Small sample size

47	Single-blind, RCT	60	Treatment arm: auricular acupuncture intervention control arm: sham auricular acupuncture intervention	Auricular acupuncture revealed a significant increase in ghrelin level and decrease in leptin level than sham auricular acupuncture	Single-blind trial Small sample size

Note: RCT: randomized clinical trial; PR: pregnancy rate; OPR: ongoing pregnancy rate; LBR: live birth rate. ^*∗*^*P*< 0.05 versus treatment arm.

**Table 2:** Summary of randomized studies of the effect of CHM on infertility outcomes.

Study ID	Design	Sample size	Interventions	Outcomes	Composition	Limitation
97	RCT	150	Treatment arm: group B : CC + Zhushi Tiaojing Cuyun formula control arm: group A : CC; group C : CC + aspirin	Treatment arm: PR, group B: 52% [26 of 50]^*∗*^ control arm: PR, group A: 18.0% [9 of 50] PR, group C: 32.0% [16 of 50]^*∗*^	Zhushi Tiaojing Cuyun formula: radix codonopsis (Dang Shen), astragalus root (huang Qi), *Angelica sinensis* (Dang Gui), prepared rehmannia root (Shu Di Huang), Morinda officinalis (Ba Ji Tian), Epimedium (Yin Yang Huo), Dodder (Tu Si Zi), Raspberry (fu Pen Zi), Photinia leaf (Shi Nan Ye), acorus tatarinowii (Shi Chang Pu), Salvia (Dan Shen), Safflower (hong Hua), Human placenta powder (Zi He Che Fen), Citrus (Chen Pi)	Not mentioned blindness and drop-out rate

98	RCT	80	Treatment arm: Bushen Peiyuan Yanggong decoction + estrogen + progesterone control arm: estrogen + progesterone	Treatment arm: PR, 57.50% [23 of 40]^*∗*^ control arm: PR, 37.50% [15 of 40]	Bushen Peiyuan Yanggong decoction: astragalus (Huang Qi), prepared rehmannia root (Shu Di Huang), Dodder (Tu Si Zi), Cyathula root (Chuan Niu Xi), Fructus Lycii (Gou Qi Zi), *Angelica sinensis* (Dang Gui), Danshen root (Dan Shen), Epimedium (Yin Yang Huo), Ligusticum wallichii (Chuan Xiong), roasted liquorice (Zhi Gan Cao)	Not mentioned blindness and drop-out rate Small sample size

100	RCT	120	Treatment arm: Yuyin Ling + clomiphene control arm: clomiphene	Treatment arm: PR, 46.7% [28 of 60]^*∗*^ control arm: PR, 20.0% [12 of 60]	Yuyin Ling : Yam (Shan Yao), prepared rehmannia root (Shu Di Huang), Chinese herbaceous peony (Shao Yao), Dodder (Tu Si Zi), *Angelica sinensis* (Dang Gui), Eucommia (Du zhong), Placenta (Zi he che), Cyperus (Xiang fu), Danshen root (Dan shen), achyranthes bidentata (huai niu xi), Tortoise shell (Gui jia), Bupleurum (Chai hu) Modifications: Severe phlegm dampness: Add Citrus (Chen pi), acorus tatarinowii (Shi chang pu) Severe blood stasis: Add Ligusticum wallichii (Chuan xiong), Trogopterus dung (Wu ling zhi), *Angelica sinensis* (Dang gui)	Not mentioned blindness and drop-out rate

102	RCT	76	Treatment arms: Huoxue Quyu formula + ciprofloxacin control arms: ciprofloxacin	Treatment arm: PR, 39.5% [15 of 38]^*∗*^ control arm: PR, 18.4% [7 of 38]	Huoxue Quyu formula: Red peony (Chi Shao), Dried ginger rhizome (Gan Jiang), Peach kernel (Tao Ren), Safflower (hong Hua), Ligusticum wallichii (Chuan Xiong), Tree peony bark (Dan Pi), Fennel (Xiao Hui Xiang), Radix aucklandiae (Mu Xiang), Herba Patriniae (Bai Jiang Cao)	Not mentioned blindness and drop-out rate Small sample size

103	RCT	80	Treatment arm: Wenjing Tongluo decoction + Tubal hydrotubation control arm: Tubal hydrotubation	Treatment arm: PR, 67.5% [27 of 40]^*∗*^ control arm: PR, 40% [16 of 40]	Wenjing Tongluo Decoction: Evodia rutaecarpa (Wu Zhu Yu), White peony root (Bai Shao), Dwarf lilyturf (Mai Dong), Ligusticum wallichii (Chuan Xiong), cassia twig (Gui Zhi), Moutan (Mu Dan Pi), hide gelatin (E Jiao), Ginger (Sheng Jiang), *Angelica sinensis* (Dang Gui), Pinellia ternate (Ban Xia), Licorice (Gan Cao)	Not mentioned blindness and drop-out rate Small sample size

106	RCT	56	Treatment arm: Bushen Culuan Decoction control arm: estradiol valerate tablets/estradiol cyproterone tablets (clement) +clomiphene	There was no significant difference in outcomes of PR between two groups	Bushen Culuan Decoction: Dodder (Tu Si Zi), Ligustrum (Nv Zhen Zi), Medlar (Gou Qi Zi), Mistletoe (Sang Ji Sheng), Radix dipsaci (Xu Duan), cyathula root (Chuan Niu Xi), Red peony (Chi Shao), *Angelica sinensis* (Dang Gui), Lycopus lucidus (Ze Lan), Danshen root (Dan Shen), Rhizoma cyperi (Xiang Fu), Cattail pollen (Pu Huang)	Not mentioned blindness Small sample size

107	RCT	80	Treatment arm: self-designed Bushen huoxue decoction + ethinylestradiol cyproterone tablets + clomiphene citrate capsules control arm: ethinylestradiol cyproterone tablets + clomiphene citrate capsules	Treatment arm: PR, 85% [34 of 40] ^*∗*^ control arm: PR, 65% [26 of 40]	Self-designed Bushen huoxue Decoction: *Angelica sinensis* (Dang Gui), Ligusticum wallichii (Chuan Xiong), Epimedium (Yin Yang Huo), Danshen root (Dan shen), Dodder (Tu Si Zi), prepared rehmannia root (Shu Di Huang), Dried radix rehmanniae (Sheng Di Huang), Red peony (Chi shao), Ligustrum (Nv Zhen Zi), Eclipta (Mo Han Lian), Cyathula root (Chuan Niu Xi), Morinda officinalis (Ba Ji Tian), Herba leonuri (Yi Mu Cao), Safflower (hong Hua), Bupleurum (Chai Hu), Licorice (Gan Cao)	Not mentioned blindness and drop-out rate Small sample size

108	RCT	80	Treatment arm: Shugan Jianpi formula + bromocriptine control arm: bromocriptine	Treatment arm: PR, 42.5% [17 of 40] ^*∗*^ control arm: PR, 17.5% [7 of 40]	Shugan Jianpi formula: bupleurum (Chai Hu), raw malt (Sheng Mai Ya), *Angelica sinensis* (Dang Gui), White peony root (Bai Shao), Indian bread (Fu Ling), atractylodes (Bai Zhu), achyranthes bidentata (Niu Xi), Licorice (Gan Cao)	Not mentioned blindness and drop-out rate Small sample size

109	Multicenter double-blind placebo parallel controlled RCT	202	Treatment arm: ①Before ovulation: Huoxue Xiaoyi granule; ②After ovulation: Bushen Zhuyun granule control arm: Placebo treatment	Treatment arm: PR, 44.6% [45 of 101] ^*∗*^ LBR, 34.7% [35 of 101] ^*∗*^ control arm: PR, 29.7% [30 of 101] LBR,20.8% [21 of 101] ^*∗*^	Huoxue Xiaoyi granule: radix bupleuri (Chai Hu), Cyperus (Xiang Fu), Salvia Miltiorrhizae (Dan Shen), Rhizoma Curcuma (Jiang Huang), Radix Paeoniaerubra (Shaoyao) Bushen Zhuyun Granule: Radix Bupleuri (Chai Hu), Indian bread (Fu Ling), Ligustrum lucidum (Nv Zhen Zi), Eclipta (Mo Han Lian), Rhizoma atractylodes (Bai Zhu) Radix dipsaci (Xu Duan)	

110	RCT	62	Treatment arm: Bushen Yangjing granule + letrozole control arm: compound packaging of estradiol tablets/estradiol and progesterone tablets + letrozole	Treatment arm: PR, 67.7% [21 of 31] ^*∗*^ control arm: PR, 35.5% [11 of 31]	Bushen Yangjing granule: prepared rehmannia root (Shu Di Huang), *Angelica sinensis* (Dang Gui), White peony root (Bai Shao), Ligusticum wallichii (Chuan Xiong), Dodder (Tu Si Zi), Fructus Lycii (Gou Qi Zi), Semen plantaginis (Che Qian Zi), the fruit of Chinese magnoliavine (Wu Wei Zi), Fructus rubi (Fu Pen Zi), Cyathula root (Chuan Niu Xi), Cyperus (Xiang Fu), Fried Fructus aurantia (Chao Zhi Qiao), Radix codonopsis (Dang Shen), Epimedium (Yin Yang Huo), Salty anemarrhena asphodeloides (Yan Zhi Mu), Herba leonuri (Yi Mu Cao) Modifications: Postmenopausal: Add the amount of Dodder (Tu si zi), and add fallopia multiflora (He Shou Wu), remove Herba leonuri (Yi Mu Cao) Intermenstrual period: Add Morinda officinalis (Ba Ji Tian), The seed of cowherb (Wang Bu Liu Xing), Liquidambar formosana hance (Lu Lu Tong); Premenopausal: Add amethyst (Zi Shi Ying), Radix dipsaci (Xu Duan); Menstrual period: Add Semen persicae (Tao Ren), Safflower (Hong Hua)	Not mentioned blindness and drop-out rate Small sample size

112	RCT	60	Treatment arm: Bushen huoxue formula control arm: oral estradiol valerate	Treatment arm: PR, 47.7% [14 of 30] ^*∗*^ control arm: PR, 20.0% [6 of 30]	Bushen huoxue formula: Bupleurum (Chai Hu), Dodder (Tu Si Zi), Raspberry (fu Pen Zi), Curculigo orchioides (Xian Mao), Psoralea (Bu Gu Zhi), prepared rehmannia root (Shu Di Huang), Epimedium (Yin Yang Huo), *Angelica sinensis* (Dang Gui), Rhizoma Dioscoreae (Shan Yao), Indian bread (Fu Ling), Ligusticum wallichii (Chuan Xiong), Cyperus (Xiang Fu), Dwarf lilyturf (Mai Dong), Roasted liquorice (Zhi Gan Cao), Parched hawthorn fruit (Jiao Shan Zha)	Not mentioned blindness and drop-out rate Small sample size

113	RCT	120	Treatment arm: Bushen Quyu decoction + laparoscopic surgery, and then gestrinone treatment control arm: laparoscopic surgery, and then gestrinone treatment	Treatment arm: PR, 68.3% [41 of 60] ^*∗*^ control arm: PR, 43.3% [26 of 60]	Bushen Quyu decoction: polygonatum (Huang Jing), fallopia multiflora (He Shou Wu), Yam (Shan Yao), Epimedium (Yin Yang Huo), prepared rehmannia root (Shu Di Huang), Ligusticum wallichii (Chuan Xiong), Dodder (Tu Si Zi), Citrus (Chen Pi), Moutan (Dan Pi), Placenta (Zi He Che), Sliced deerhorn (Lu Jiao Pian)	Not mentioned blindness and drop-out rate

114	RCT	70	Treatment arm: Jinlinzi powder + sini powder + conventional western medicine control arm: conventional western medicine	Treatment arm: PR, 82.86% [29 of 35] ^*∗*^ control arm: PR, 60% [21 of 35]	Jinlinzi powder + Sini powder: Jinlingzi, Bupleurum (Chai Hu), Radix aucklandiae (Mu Xiang), White peony root (Bai Shao), fruit of citron or trifoliate orange (Zhi Shi), Corydalis tuber (Yan Hu Suo), Inner layer of cinnamon (Gui Xin), Roasted liquorice (Zhi Gan Cao)	Not mentioned blindness and drop-out rate small sample size

115	RCT	60	Treatment arm: Jianpi Bushen Zhuluan formula + letrozole control arm: letrozole	Treatment arm: PR, 56.7% [17 of 30]^*∗*^ control arm: PR, 30% [9 of 30]	Jianpi Bushen Zhuluan formula: Dodder (Tu Si Zi), Radix codonopsis (Dang Shen), Dried radix rehmanniae (Sheng Di Huang), Yam (Shan Yao), prepared rehmannia root (Shu Di Huang), Lotus fruit (Lian Zi Rou), Radix scutellariae (Huang Qin), Radix glehniae (Bei Sha Shen), Dendrobe (Shi Hu), polygonatum (huang jing), rose (Mei Gui Hua), Sargentodoxa cuneata (Hong Teng), Citrus (Chen Pi), Tangerine leaf (Ju Ye)	Not mentioned blindness and drop-out rate Small sample size

Note: RCT: randomized clinical trial; PR: pregnancy rate; LBR: live birth rate. ^*∗*^*P*< 0.05 versus treatment arm.

**Table 3:** Clinical studies on retention enema in infertility treatment.

Study ID	Design	Sample size	Interventions	Outcomes	Limitation
146	RCT	92	Treatment arm: penqiangyan prescription and TCM retention enema control arm: routine treatment	Treatment arm: TE 80.43%^*∗*^ control arm: TE95.65%	Not mentioned drop-out rate

147	RCT	53	Treatment arm: hysteroscopy and TCM retention enema control arm: hysteroscopy	Treatment arm: PR 55.56%^*∗*^ control arm: PR23.08%	Not mentioned drop-out rate Small sample size

148	RCT	50	Treatment arm: laparoscopic surgery and TCM retention enema control arm: laparoscopic surgery	Treatment arm: PR72%^*∗*^ control arm: PR44%	Not mentioned drop-out rate Small sample size

149	RCT	86	Treatment arm: hysteroscopy and TCM retention enema control arm: hysteroscopy	Treatment arm: PR 62.7%^*∗*^ control arm: PR41.8%	Not mentioned drop-out rate

150	RCT	98	Treatment arm: tubal interventional recanalization and TCM retention enema control arm: tubal interventional recanalization	Treatment arm: PR53.1%^*∗*^ control arm: PR20.4%	Not mentioned drop-out rate

153	RCT	60	Treatment arm: TCM retention enema, acupuncture and estradiol tablets control arm: estradiol tablets	Treatment arm: TE 83.3%^*∗*^ control arm: TE50.0%	Not mentioned drop-out rate Small sample size

Note: RCT: randomized clinical trial; PR: pregnancy rate; TE : total effective rate; ^*∗*^*P*< 0.05 versus treatment arm.
